# Satellite imaging data analysed to evaluate the effectiveness of land-use zoning for the protection of built heritage at Bagan, Myanmar

**DOI:** 10.1016/j.dib.2019.104701

**Published:** 2019-10-21

**Authors:** Benjamin Edwards, Tilman Frasch, Julia Jeyacheya

**Affiliations:** Manchester Metropolitan University, United Kingdom

**Keywords:** Land-use, Zoning, Myanmar, World heritage, Satellite remote-sensing, Archaeology, Buddhism

## Abstract

This data in brief article describes the data collection and analysis process undertaken to assess the effectiveness of land-use zoning for heritage protection at the UNESCO World Heritage Site of Bagan Myanmar [1]. In order to measure the expansion of urban areas within the Archaeological Zone of Bagan from 1987 to 2018, and thus assess whether development controls functioned effectively, imaging data was acquired from the archives of the US Landsat 5, and the European Space Agency's Sentinel 2 platforms. Shapefiles were generated in QGIS for each settlement within the zone for each of the eight sample years. The changing area, in square kilometres, of these settlements was then analysed over time, with further reference to two ‘control’ settlements that exist outside of the Archaeological Zone.

**Table:** Specifications Table

Subject	Archaeology
Specific subject area	Satellite remote sensing data as an aid to heritage management.
Type of data	Table Chart Shapefile
How data were acquired	Satellite image data was downloaded from the Landsat 5 database provided by the US Geological Survey, and the European Space Agency Copernicus Sentinel 2 database
Data format	Analysed
Parameters for data collection	Satellite data was required for five-yearly intervals from 1987 to 2018, with allowance made for satellite availability and cloud cover; the images had to be acquired during the Myanmar dry season; cloud cover had to be zero.
Description of data collection	Landsat and Sentinel 2 data are freely available to researchers from the US Geological Survey and ESA Copernicus online databases. These were downloaded and then subject to classification QGIS, which generated the data tables and shapefiles provided in this article.
Data source location	Institution: US Geological Survey - https://www.usgs.gov/land-resources/nli/landsat Institution: European Space Agency
Data accessibility	With the article
Related research article	Edwards, B; Frasch, T & Jeyacheya, J. Evaluating the effectiveness of land-use zoning for the protection of built heritage in the Bagan Archaeological Zone, Myanmar. A satellite remote-sensing approach *Land Use Policy*

**Table:** 

**Value of the Data** • These data demonstrate the efficacy of manual ground cover classification to produce meaningful analytical results, without the need for training automatic or semi-automatic classification algorithms, which may be beyond the capacity of individual researchers • These data outputs could be used as exemplars for researchers designing ground-cover classification workflows • The data and/or the method can form the basis for wider studies of urban expansion in ecologically or culturally sensitive landscapes • When used in conjunction with the quoted and freely-available satellite coverages, the data could be used to assess the utility of satellite datasets of differing age and quality (i.e. Landsat, versus ESA) to produce meaningful conclusions for a variety of different planning applications

## 1 Data

Data was required to assess the effectiveness of land-use zoning for heritage protection, aimed to prevent urban expansion within the Archaeological Zone of Bagan, Myanmar [1]. Newly inscribed on the UNESCO World Heritage List, Bagan comprises some 3388 Buddhist monuments, with the earliest dating to the 9th and 10th centuries CE. The setting of these monuments could be compromised by urban expansion, and thus the Myanmar government delineated different ‘archaeological zones’ over the landscape surrounding Bagan. These covered the entire 60 sq. km area within which archaeological sites and monuments are located, and therefore at risk of urban development. To assess the effectiveness of the development controls, data was extracted from satellite imaging from 1988 to 2018. Ten shapefiles are provided with the article, packaged as *.zip files, which contain area-type coverages for each of the settlements that fall within the Bagan Archaeological Zone, and for two ‘control’ settlements (Chauk and Pakokku) that are outside the zone to the south and north, respectively.

The shapefiles for the urban settlements within the Bagan Archaeological Zone are organised by year, and the associated data table within each file has an entry for each individual settlement within the zone, numbering 19 settlements in total. Each settlement also has an ‘area’ entry, in square kilometres, in each year's associated data table (see below). The shapefiles for the settlements within the Bagan Archaeological Zone have two different prefixes, depending on the satellite data source from which the shapefiles were generated, followed by a six-figure value representing the day/month/year that the satellite data was captured. So, those six ZIP files prefixed ‘Bagan L’ contain shapefiles generated from Landsat data, provided by the US Geological Survey, which was captured between 28th December 1987 and 28th January 2011. Two ZIP files with the prefix ‘Bagan S2’, followed by the date, contain shapefiles derived from Copernicus Sentinel 2 data provided by the European Space Agency, from 2015 to 2018. Each of these shapefiles have associated data tables that provide the name of each settlement within the zone, a unique identifier, and the area that the settlement covered in that year (in square kilometres).

The remaining two ZIP files, ‘Chauk’ and ‘Pakokku’, also contain shapefiles, but in these instances the associated data tables are organised differently, because each of these files represent a single settlement, rather than the 19 contained within each Bagan shapefile (above). In the Chauk and Pakokku files the data tables have an entry for each year in which area data was generated for the settlement. Thus, each shapefile contains area data for the size of the settlement from 1987 to 2018 at roughly five-yearly intervals, comprising an area-type vector coverage, an identifier, and a square kilometre value.

The final data file, entitled ‘Settlement Area Data.xlsx’ contains the aggregated square-kilometre area data for all of the Bagan Archaeological Zone settlements, Chauk and Pakokku. It also contains data tables and graphical representations of the change in the settlement areas over time, including percentage changes in settlement area between each sampled year.

## 2 Experimental design, materials, and methods

In order to measure the change of urban settlement size over time, both within the Bagan Archaeological Zone and in the two control settlements of Chauk and Pakokku, satellite imaging data was acquired from 1987 to 2018, at roughly five yearly intervals. The specific date of acquisition for each data point was flexible, as it relied upon the availability of consistent satellite coverage for the entire area, and on a zero-cloud cover value – usually during the dry season corresponding to northern hemisphere winter. The start date of 28th December 1987 was chosen to begin the analysis, as this was the closest available that fulfilled the above conditions to the 1988 commencement of the Bagan Symposium, when heritage policy began to be formed as regards the Buddhist monuments in the area.

Two satellite platforms were chosen to provide the earth-observation data necessary for the analysis. For the first six data points - of 1987, 1992, 1997, 2001 and 2007 - Landsat 5 was the most suitable freely available photographic satellite platform, with a ground resolution of 30 m per pixel. For the data points of 2015 and 2018, the European Space Agency's Sentinel 2 satellite platform became the most suitable source, as it benefits from a ground-resolution of 10 m per pixel [3]. Landsat data is provided by the US Geological Survey, and Sentinel data through the ESA's Copernicus programme. Both services allow the selection of coverage areas anywhere on the earth and provide fully georeferenced *.JP2 files for use in Geographic Information Systems (GIS) software, based on the WGS84 coordinate system.

The image files for each data point were then imported in the QGIS package. Data from both satellite platforms cover the visible light spectra and several near-visible bands, thus the first processing step was to combine the RGB bands to produce a true-colour image suitable for analysis. For Landsat 5 data these RGB bands are 3, 2 and 1, whilst for Sentinel 2 they are bands 4, 3 and 2, respectively. Prior to the classification of urban from non-urban ground coverage, it was also necessary to ensure the displayed values of these bands was consistent across the different images. In both cases the images were rendered as multiband colour; for the Landsat 5 images the RGB values were as follows: R = 0–66; G = 0–54; B = 0–115. For the Sentinel 2 images, the RGB values were as follows: R = 384–2041; G = 639–1758; B = 811–1667.

When the consistent display of the image data was achieved, the classification of modern urban from non-urban areas was undertaken manually. The analysis was not concerned with the expansion of individual farmsteads within the archaeological zones at Bagan, but with the expansion of urban settlements, which should be controlled under the Township Law and Order Council for Pagan-Nyaung-Oo Township [2]. An urban area was defined and identified as a series of buildings grouped together as a recognisable settlement, in most cases associated with a township name, although in two cases names could not be determined from available maps of Myanmar, which are of indifferent quality. The limits of settlements were defined as the end of contiguous development, and thus a settlement as a whole represented the uninterrupted presence of buildings. There was also a pragmatic, if not initially designed, minimum size for a settlement based on the resolution of the satellite imagery – the ground resolution of each pixel ensured that no area of structures could go below the 10 m resolution of Sentinel data, or the 30 m resolution of Landsat data. In practise all settlements were comfortably larger than these minimum values, and no indeterminacy entered the analysis as a result (see square kilometre data in the associated data).

In order to measure the area of the 19 settlements within the Bagan Archaeological Zone and the two control settlements of Chauk and Pakokku, ‘surface’ or ‘polygon’ type shapefiles were created by manually tracing the limits of each urban area. This was undertaken for each yearly data point, and each shapefile attribute table contains an entry for each named urban settlement. In addition to this manually generated data, the QGIS plugin ‘autofields’ [4] was used to add area data, in square kilometres, for each settlement. The shapefile attribute tables for each year's data point were exported in CSV format for analysis in Microsoft Excel.

Analysis of the data concerned the change in absolute size of each settlement within the Bagan Archaeological Zone and the controls of Chauk and Pakokku, and rate of change between each data point, expressed as a percentage. It was here that the control settlements were important to the analysis. Two settlements outside of the Archaeological Zone were vital to assess whether the development controls within the Zone were efficacious in limiting development or otherwise.
